# Canine Behavioral Assessment and Research Questionnaire (C-BARQ): Validation of the Italian Translation

**DOI:** 10.3390/ani13071254

**Published:** 2023-04-05

**Authors:** Anna Broseghini, Cécile Guérineau, Miina Lõoke, Chiara Mariti, James Serpell, Lieta Marinelli, Paolo Mongillo

**Affiliations:** 1 Department of Comparative Biomedicine and Food Science, University of Padua, Viale dell’Università 16, 35020 Legnaro, PD, Italy; 2 Department of Veterinary Sciences, Università di Pisa, Viale delle Piagge 2, 56124 Pisa, PI, Italy; 3 School of Veterinary Medicine, University of Pennsylvania, 3900 Delancey Street, Philadelphia, PA 19104, USA

**Keywords:** aggression, anxiety, behavioral problems, dog, questionnaire

## Abstract

**Simple Summary:**

A large percentage of dogs expresses problematic behaviors that can be explored using questionnaires. However, due to the subjectivity of owners’ replies, before their use in research, questionnaires should undergo a process evaluating their reliability and validity. This is also necessary when an existing and valid questionnaire is translated into a different language. The aim of this study was to validate an Italian translation of the widely used Canine Behavioral Assessment and Research Questionnaire (C-BARQ), originally developed in English. The statistical analysis showed that 62 of the 100 questionnaire items could be grouped into 13 factors, each underlying a category of dog behaviors, as expected from the original structure of C-BARQ as well as from previous research using this tool. The main differences between the Italian factorial structure and that of the most recent English version regarded: items related to stranger-directed fear and aggression, which are represented by two separate factors in the English version, were grouped in a single factor in the Italian one; the factor Dog rivalry, present in the English version but not in the Italian one; and factors Dog-directed aggression and Touch sensitivity, which emerged in our analysis, but are not present in the English one. In spite of these differences, there is large overlap between our factorial structure and that of studies using C-BARQ in other languages, indicating that the 62-item Italian version presented in the current study can be reliably used in research.

**Abstract:**

The aim of this study is to develop an Italian translation of the 100-item Canine Behavioral Assessment and Research Questionnaire (C-BARQ) version and to validate its psychometric properties, in order to facilitate systematic, large-scale studies on dog behavior for Italian-speaking dog owners. A total number of 803 responses by dog owners were collected online. Using the Principal Axis Method and Common Factor Analysis with Quartimin oblique rotation (*p* < 0.05), a factorial structure was found including 13 factors composed of 62 items and explaining 53.5% of the total variance. Eight factors showed high reliability (Cronbach’s alpha > 0.70), namely: stranger-directed aggression/fear, dog-directed fear, owner-directed aggression, separation-related behavior, chasing, dog-directed aggression, attachment/attention seeking, and elimination problems. Three factors were slightly under the threshold and two had only modest reliability (non-social fear, energy level, touch sensitivity, excitability and trainability). A potential explanation for factors with low reliability is that the composing items do not describe behaviors resulting from homogeneous stimuli or situations. Although our factorial structure resembled in most respects that of the most recently published Canadian version, some important exceptions are present regarding dog rivalry, intraspecific aggression, fear/aggression towards strangers, touch sensitivity and chewing inappropriate objects. Such differences may be due to demographic and/or cultural differences between the sampled populations. Overall, the results suggest that a 62-item Italian C-BARQ can be reliably used in studies on dog behavior.

## 1. Introduction

A large percentage of dogs is reported to express problematic behaviors. For example, a recent survey on more than 4000 dogs revealed that the prevalence of canine behavior problems was as high as 85% [1]. Another study reports that 72.5% of pet dogs in Finland displayed some highly problematic behavior, including noise sensitivity, fears, separation-related behavior, inattention, aggression, hyperactivity/impulsivity, and compulsive behavior [2]. In the stricter meaning of the term, problematic behaviors are the outward manifestations of pathological and maladaptive conditions, such as, for instance, generalized anxiety disorder or cognitive dysfunction syndrome [3]. These conditions have obvious, negative implications for the dog’s health and welfare. However, some putative behavioral problems are often behaviors potentially normal for the dog in a given context but deemed as undesirable or problematic by the owner, such as barking, digging, running after other animals, etc. It was estimated that up to 90% of dogs express behaviors that are seen as unacceptable by their owners [4]. Even if non pathological, these problematic behaviors can also have relevant negative implications for the dogs’ welfare, as they threated the dog-owner relationship. For instance, behavioral reasons for relinquishment in shelters include potentially non pathological problematic behaviors, such as house soiling, disobedience and vocalizations [5,6]. Regardless of the distinction into pathological and undesirable behaviors, both are a frequent cause of canine abandonment [5], relinquishment to shelters [7] and euthanasia [8,9].

Considering the pervasiveness of behavioral problems and the potentially severe effects they have on the animal’s welfare, it would be relevant to perform systematic studies aimed to understand their causes, consequences, and possible interventions [10]. Therefore, suitable methods are needed to help identify and characterize dog behaviors, especially those that deviate from normality.

Different ways have been developed to characterize dog’s behavior [11]. Methods involving the direct observation of the animal, such as test batteries, ratings of individual dogs by experts, and observational tests [12] have the advantage of being generally accurate, controlled, and producing relatively objective data. However, they also have disadvantages, such as being time and resource consuming, and not allowing the systematic assessment of many dogs. Moreover, dogs are often observed outside their usual environment and only for short intervals of time, which makes it difficult to detect behaviors that occur rarely and only under specific circumstances. One possibility to bypass such shortcomings is to rely on information provided directly by the owner. It is reasonable to assume that the owners are the observers who know best about their dog’s behavior. Although owners might not be educated observers and their perceptions influenced by the relationship with the dog and by personal expectations, the daily sharing of time and space with that individual dog might make the owners skilled in recognizing their dog’s behavioral characteristics and possible changes [13]. One of the most widely used tools for collecting information directly from owners or caretakers are questionnaires. The use of questionnaires to describe an individual’s behavioral characteristics is growing in popularity, both in human personality research (e.g., [14]), as well as in research with companion animals, (e.g., [15,16,17,18]).

Questionnaires seem to be a suitable tool for studying dog behavior in a systematic and large-scale manner. As data about behaviors collected through questionnaires involve a subjective component, it is necessary to first establish their reliability and validity. Generally speaking, reliability is the extent to which a measurement is repeatable and consistent; for example, a test or a questionnaire can be considered reliable if the repeated measurement under constant conditions provides the same result [19]. When applied to questionnaires, a specific measure of reliability is the so called ‘internal consistency’. This parameter indicates the extent to which the items of the questionnaire, or items that compose a specific factor, measure the same underlying construct. Assessment of the internal consistency is most commonly completed via calculation of the Cronbach’s Alpha coefficient [20]. Validity is also a multi-faceted term, that overall refers to how well the questionnaire measures what it is meant to measure [19]. There are several types of validity that apply to questionnaires. Face validity defines how well a measure appears to be related to a specific construct in the opinion of experts [20]. Content validity is the degree to which items in an instrument reflect the content universe to which the instrument will be generalized [21]. Construct validity, with its two component, discriminant and convergent validity, refers to how well the concept that is meant to be measured is operationalized into concrete and measurable characteristics, the verification of which is often done by factor analysis [20]. Finally, criterion validity is the degree to which a measure is related to an outcome [20]. That being said, it is important that studies aiming to investigate dog behavior rely on valid and reliable instruments.

The Canine Behavioral Assessment and Research Questionnaire (C-BARQ), developed by Hsu and Serpell in 2003, is a questionnaire aimed at investigating normal and abnormal behaviors in dog populations, whose validity and reliability have been extensively assessed. The questionnaire was originally composed of 11 factors and used to screen behavioral traits of companion dogs in the USA [22]. In 2012, Duffy and Serpell added 3 additional factors to create the current version of the C-BARQ comprising 100 items, grouped into 14 factors [23]. In recent years, the C-BARQ has been used in numerous studies, for example, to classify behavioral phenotypes in genetic and morphological studies [24,25,26,27,28], to assess behavioral changes or problems [29,30,31], to evaluate temperament in guide and service dogs [23], and also to assess dogs’ performance in problem solving tasks [15]. As already mentioned above, the validity and reliability of the C-BARQ instrument have been extensively assessed. The structure of the questionnaire was verified through the factor analysis. Internal reliability of the factors was found equal or greater (Cronbach’s alpha ≥ 0.70) than the generally accepted threshold [32], the test–retest reliability of the factors ranged from r = 0.25 to 0.56, with an average correlation coefficient of r = 0.47, and the average percentage agreement (quadratic weighted formula) for the items composing each factor ranged from 82% to 97% [33]. Moreover, discriminant and convergent validity were assessed determining that the results of the factor analysis matched with particular behavioral diagnoses made by external experts. More details about the test reliability and the process of validation can be found in Hsu & Serpell, 2003 [22].

The C-BARQ instrument has also caught the attention of scientists wishing to use it with non-English-speakers. However, the validity of a questionnaire may not necessarily apply for people who are non-native speakers of the language, or residing in a country different from the one in which the questionnaire was developed and validated. In the specific case of the C-BARQ, relevant socio-cultural differences may exist among countries, for instance in the perception of the dog’s role by both society and individual owners, in the educational background of professional figures, in the approaches to dog management and training, and in the interpretation/acceptance of different dog behaviors. Under these premises, the C-BARQ was translated into different languages, and the validity of the translation experimentally verified. The list of languages (Countries) in which the questionnaire has been translated include Mandarin Chinese (Taiwan) [31], Japanese (Japan) [34], Farsi (Iran) [35], Spanish (Mexico) [36], Portuguese (Portugal) [37], Swedish (Sweden) [13], Dutch (Netherlands) [26], Portuguese (Brazil) [38]. Regarding Italian, Marshall-Pescini and colleagues in 2008 conducted a study in which some factors of the original C-BARQ (trainability, non-social fear and stranger fear) were translated [15]. Similarly, Dalla Villa and colleagues used translations of some of the original C-BARQ factors to validate the Socially Acceptable Behaviour (SAB) test in 2016 [39]. However, a complete and valid Italian translation of the C-BARQ is missing. The Italian dog population consists of almost 9 million individuals, which makes it one of the most numerous among European countries [40], and 40.2% of Italians own a pet, of which 43.6% are dogs [41]. The consistency of the canine population, as well as the potential differences between Italy and other countries in which the C-BARQ was validated, suggest that a comprehensive tool in Italian for large-scale behavioral screening is needed.

Therefore, the aim of this study was to develop an Italian translation of the complete (100 items) C-BARQ version [23] and to validate its psychometric properties, in order to use it for Italian-speaking dog owners, to facilitate systematic, large-scale studies on dog normal and problem behavior.

## 2. Materials and Methods

### 2.1. Questionnaire

The questionnaire used in the current study is an Italian translation of the C-BARQ developed by Duffy and Serpell [23]. The questionnaire was translated into Italian by one of the authors and subsequently reviewed to improve the appropriateness of the translation by three designated experts: two senior scientists in the field of dog behavior, and a veterinarian specialist in behavioral medicine. Afterwards, an independent professional translator was asked to back-translate the Italian C-BARQ questionnaire into English, in order to assess its correspondence with the original C-BARQ. After completion of this step, the questionnaire was administered to the participants. The questionnaire consists of 100 items, divided in 14 sub-scales, about dog’s behavioral responses to a variety of everyday situations, during the recent past. Owners were asked to rate their dogs’ responses on a series of 5-point Likert scales of frequency (from 0 meaning “never” to 4 meaning “always”) or of intensity (from 0 meaning “no sign of the behavior” to 4 meaning “severe form of the behavior”), depending on the item. Participants were invited to answer to all questions; if participants had never observed their dog in the described situation or they did not feel able to answer, the option “Not observed/not applicable” was available, to which no score was attributed. In addition to the 100 C-BARQ questions, the Italian questionnaire collected demographic information of both the respondent, such as gender, age, occupation, length of ownership, ownership of how many dogs, and the dog, such as sex, age, breed, reproductive status. The administered Italian questionnaire is available in the Supplementary Materials.

### 2.2. Participants and Data Collection

The Italian version of the questionnaire was distributed among Italian citizens, owners of a dog for at least 6 months at the time of compilation. No other criterion was set for participation, therefore, any dog of at least 6 months of age was eligible. If the participants owned more than one dog, they were asked to select only one of the dogs and answer the questions referring to the behavior of the chosen one. The questionnaire was made available online and shared via professional and personal contact network of the authors and through social networks. The questionnaire remained accessible for about 3 months and the questionnaire responses collected in this period of time were used for the analysis.

### 2.3. Statistical Analysis

An exploratory factor analysis was first performed to aggregate data into a smaller set of variables. Data were analyzed in JMP Pro software (Version 16.2.0, SAS Institute Inc., Cary, NC, USA, 2021) using the Principal Axis Method and Common Factor Analysis, with Quartimin oblique rotation [42]. For items with less than 25% of missing responses JMP’s Automated Data Imputation Tool was used to impute missing data for the remaining variables. Items with more than 25% missing data were excluded from the analysis. Because all four items referring to aggressive behavior towards other dogs living in the same household fell below this threshold (see Results), all the items in the “Familiar Dog Aggression” factor were eliminated. As a consequence, the factor analysis was restricted to the remaining 96 items in the original C-BARQ. Only the items with factor loadings ≥ 0.4 or ≤−0.4 were considered. In case one item had a factor loading ≥ 0.4 or ≤−0.4 in more than one factor, it was assigned to the factor where it had the highest loading. Finally, Cronbach’s alpha was calculated to assess the internal consistency of the factors. Factors with Cronbach’s alpha values ≥ 0.70 were considered reliable.

## 3. Results

A total number of 803 responses to the questionnaire were collected. The missing rate of total data ranged from 0% to 68.5% (mean 6.0 ± 13.8%, median 1.5%). Items number 32 (“The dog acts aggressively towards another (familiar) dog in your household”), 33 (“The dog acts aggressively when approached at a favorite resting/sleeping place by another household dog”), 34 (“The dog acts aggressively when approached while eating by another household dog”) and 35 (“The dog acts aggressively when approached while playing with/chewing a favorite toy, bone, object by another household dog”) had missing data rate greater than 25%, and therefore were removed. The missing rate of the remaining items ranged from 0% to 24%.

### 3.1. Factor Analysis

The structure resulting from the Factor Analysis explained 53.5% of the total variance. The results of the factor analysis with the loading value for each item in the 13 factors are reported in Table 1. Out of the 96 items analyzed, 62 had a loading value ≥ 0.4 on at least one factor. The results of the factor analysis were similar to the factorial structure of the most recent Canadian version from Flint and colleagues [43], with few relevant differences. In the Italian analysis, the factor “Dog rivalry/Familiar dog aggression”, found in Flint et al. [43], was not included. The Italian analysis extracted a “Dog-directed aggression” factor, that was also detected in the original version of the C-BARQ [22] but not found in version by Flint and colleagues [43]. In the present analysis, the items forming the factors “stranger-directed aggression” and “stranger-directed fear” in the Canadian version [43], loaded onto the same factor, which was renamed “stranger-directed aggression/fear”. Our analysis revealed a factor labeled “Touch sensitivity”, which was labeled “pain sensitivity” in the original version [22] but was not present in the version by Flint and colleagues [43]. Item number 80 (Dog chews inappropriate objects) loaded onto the “Energy level” factor of our analysis but not in any of the North American factor analyses [22,23,43].

### 3.2. Internal Consistency

Cronbach’s alpha values for the assessment of the internal consistency of the factors are reported in Table 2. Most of the factors showed good reliability (≥0.70), three were slightly under the accepted threshold, while two factors were clearly further from the threshold [32].

### 3.3. Population

Demographic characteristics of the sample of dogs and respondents in this study are shown in Table 3.

## 4. Discussion

In this study we developed an Italian translation of the most recent C-BARQ version [23] and evaluated its psychometric properties through exploratory factor analysis and analysis of internal consistency (Cronbach’s alpha coefficient). The factorial structure included 13 factors, composed by 62 items, explaining the 53.5% of the total variance. Eight factors showed high reliability, with a Cronbach’s alpha value higher than the threshold generally considered indicative of good reliability (0.70), three were slightly under the threshold and two were clearly further from the threshold [32].

A potential explanation for the low reliability of some factors is the small number of composing items. Most of the factors falling under the 0.70 threshold of alpha consisted of only three items. It is known that factors comprising fewer items tend to have lower Cronbach’s alpha values [44]. Another potentially contributing explanation includes the fact that the items composing the factors with low alphas did not describe behaviors resulting from homogeneous stimuli or situations. For instance, the factor “Nonsocial fear” refers to several different stimuli causing fear-related behavioral responses, namely sudden or loud noises, wind or wind-blown objects, and heavy traffic. On the one hand, it is known that fear of one class of stimuli often co-occurs with that of other stimuli (see, e.g., [16,45]), possibly reflecting a fear-proneness personality trait, and justifying the loading of the items onto the same factor. On the other hand, the situations and fear triggers described by these items are relatively specific and diverse. It is reasonable to assume that many dogs could be frightened in only one of these situations and not necessarily in the others, thereby contributing to the low consistency of the factor. Similar logic could be applied to “Excitability”. As a factor, the items could reflect an excitability personality trait, but it is not unreasonable that dogs might show different extents of excitation in response to the specific situations depicted by the included items, i.e., before a walk, before a car ride, or when the doorbell rings. The same applies to the factor “Energy level”, where items do not refer to a coherent and specific situation; and “Touch sensitivity”, in which not all the described actions may be equally aversive to dogs. Finally, the factor “Trainability” included two items which describe learned behavioral responses (i.e., to sit and to come back when called), two items describing a propensity for learning (slow to learn new tricks and to respond to corrections), one reflecting the dog’s attention to the owner and one the dog’s propensity to escape from home. While these items might all partly influence the perceived dogs’ trainability or ‘obedience’, they are also likely to be individually affected by other dimensions of the dog’s behavior, history and personality. Thus, the Cronbach’s alpha values of these factors were most likely affected by a relatively low coherence among the items constituting these factors. Conversely, all the factors with higher internal consistency were composed of items which describe behavioral responses to more coherent and specific situations.

Our factor analysis produced a factorial structure, i.e., number of factors and factor composition, resembling that of the original C-BARQ [22], the revised US version [23], and other translated questionnaires [31,34,35,36,37,38]. Moreover, the Italian version of the C-BARQ is very similar in structure to the most recently published Canadian version by Flint and colleagues [43], although with some important exceptions. Of relevance to this discussion, it needs to be stressed that the choice of using Flint and colleagues’ work to compare and explain the current results is dictated by two main reasons, beyond recency. First, the study results include the factorial structure with the loadings of all items, allowing detailed comparison with our results. In addition, the sample of participants in the Canadian study consisted of ordinary dog owners who filled out the questionnaire for their personal dogs, similarly to our sample of participants, again improving the appropriateness of comparisons. Conversely, in earlier studies using the US C-BARQ, a large portion of the dog sample consisted of dogs belonging to specific breed clubs [22], dogs with overt behavioral problems [22], and service/guide dogs [23].

One of the main differences between the two studies regarded factors linked to dogs’ sociality towards other dogs. In particular, the factor “Dog rivalry”, found in Flint and colleagues [43], was not included in our factor analysis due to a preponderance of missing values. Only 32% of respondents in our sample reported owning more than one dog, a percentage in line with previous studies indicating between 33% and 34% of multi-dog Italian households [46,47]. We also extracted a “Dog-directed aggression“ factor that did not emerge as a distinct factor in the paper by Flint and colleagues [43]. The lack of a “Dog-directed aggression” factor seems a peculiarity of the study by Flint and colleagues. This factor was identified in many other studies [26,31,34,36], including the original C-BARQ [22] and its revised version [23]. Flint and colleagues highlight that most of the items composing the factor were removed from the final factorial structure due to cross-loading. In turn, they tentatively explain the lack of clear loading onto a single factor by a sampling bias which favored dogs living in multi-dog households, in turn resulting in the increased presence of dogs that are sociable toward conspecifics. As reported earlier, this was clearly not the case with our sample, reflecting a characteristic of the Italian population. This explanation would therefore be consistent with our results, in which we did not find any cross-loading of these items with other factors (in fact, loadings onto other factors were extremely low for these items).

Further differences were found in the domain of dogs’ responses to human strangers. Specifically, items included in the “Stranger-directed fear” and “Stranger-directed aggression” of the US C-BARQ loaded onto the same factor in our analysis, prompting us to re-label this factor “Stranger-directed aggression/fear”. This result suggests that our respondents were not able to distinguish fearful and aggressive behaviors of the dog towards other people. The misinterpretation of dog fear and aggression by the average owner is not surprising and may be based on the fact that some behavioral components and postures, such as barking, growling, muscle tension, are common to both fearful and aggressive states, and sometimes the dog’s behavior could express both emotions (e.g., fear-induced aggression [48]). Indeed, difficulties in distinguishing between fear and aggression were identified previously, in both C-BARQ and non C-BARQ-based studies. For instance, in the Japanese C-BARQ translation study, an intermix between aggression and fear items was found for both stranger-directed and owner directed behaviors [34]. In the Portuguese translation study, dog-directed fear and dog-directed aggression loaded onto the same factor [37]. The difficulty in discriminating these behaviors is also highlighted by the fact that experience with dogs—e.g., among those who work professionally with dogs—is a crucial factor in recognizing behaviors related to fear and aggression [49,50]. Along the same lines, works by both Flint and colleagues [51] and Jacobs and colleagues [52], report that having advanced knowledge of dog behavior makes owners significantly better at behavior identification. Both of the latter studies were conducted in North America and the percentage of owners reporting themselves as having advanced knowledge of dog behavior was in both cases around 31%. Although we did not specifically collect this information, only about 2% of our respondents were found to have professional experience in the dog field. It is therefore possible that the different factorial structures in our result and those of Flint and colleagues reflect a difference in the average level of dog experience among the sampled populations.

Interestingly, the same inability did not emerge for intraspecific aggression and fear, as our factorial structure clearly resulted in two distinct factors, “Dog-directed fear” and “Dog-directed aggression”. One potential explanation lies in the higher variability of responses shown by dogs toward a person, which may often include a mix of aggressive and fearful behaviors. Conversely, dogs’ reactions toward conspecifics are likely to be more clearly characterized by one of the two components, i.e., fear/avoidance or aggression, making the two behaviors more easily discriminable. Indeed, in a retrospective analysis of dog-dog and dog-human cases of aggression, Notari and colleagues found that most of the cases of aggression toward people were defensive aggression, while most of those towards conspecifics were offensive aggressions [53]. The fact that defensive aggression, in which fear components are likely to be mixed with aggressive components, accounts for the prevalence of aggression toward people but not toward other dogs, may explain why the Italian respondents were better able to discriminate between the two behaviors when expressed toward another dog, but not when expressed toward people.

The factor “Touch sensitivity” emerged in our factor analysis but not in Flint and colleagues or some of the other translated versions of the C-BARQ [34,35,43]. However, this factor was identified in the original C-BARQ and in its revised version [22,23], as well as in the Mexican version [36], the Taiwanese and Dutch versions [26,31], and in the European-Portuguese version [37] (though sometimes referred to as “pain sensitivity”). The items included in the factor refer to grooming and bathing procedures that owners can only experience if they perform these practices themselves and do not rely on grooming or veterinary services. However, recourse to grooming services may be more common in some countries than others, or it might be limited to specific types of dogs or to owners with sufficient financial means. Thus, it is likely that the presence/absence of this factors reflects such socio/cultural differences among countries.

Finally, a last relevant difference between our results and the work by Flint and colleagues regarded the item “dog chewing inappropriate objects”. In our structure, this item loaded onto the factor “Energy level”, while in the Canadian C-BARQ it is not present in any factor. This item could reflect one of the ways in which active dogs deal with boredom [54]. Indeed, it is not hard to think that a dog who is energetic, playful and active could easily redirect some energy to objects. It is not immediately obvious why this item did not emerge in the Canadian questionnaire. However, a recent survey of chewing behavior in dogs found out that 94% of the respondents provided their pets with eatable chewing objects and they believe that chewing helps to keep the dog calm, busy, distracted from aversive experiences and prevents boredom [55]. Although, to the best of our knowledge, there is no specific data relating to Italy, it is possible that Italian owners do not provide chewable objects to their dogs to the same extent as north America owners, in turn increasing the possibility of observing the behavior as part of the “activity level”’ factor in Italy. In addition, dog chewing inappropriate objects is often reported in the literature (e.g., [56,57,58]) as a symptom of separation anxiety. However, this was clearly not the case of our results, where the item did not load onto the ‘separation-related behaviors’ factor. One possible explanation lies in the fact that the owners’ observation of this behavior occurs at a different time than the observation of other separation-related behaviors. Indeed, being restless and agitated, whining, barking and howling, shaking, shivering and salivating, are all behaviors that can be either directly observed by the owner when about to leave or possibly expressed during the owner’s absence and reported by a neighbor. Conversely, the outcome of “dog chewing inappropriate objects” is more likely detected by owners when they return home, when most other separation-related behaviors may have ceased.

## 5. Conclusions

The Italian translation of the C-BARQ presented in this paper resulted in a factorial structure that resembled in many respects that of the most recently published Canadian version, as well as that of translations in other languages. Overall, this indicates that the 62-item Italian C-BARQ can be reliably used for studies on dog behavior in Italy.

Despite the large overlap between our results and those from North America, there were also some relevant differences, which mirror differences in demographic characteristics, and possibly cultural differences. In particular, the complete absence of a ‘Dog rivalry’ factor can be attributed to the limited presence of multi-dog households in our sample. Extending the administration of the questionnaire to a targeted sample of multi-dog owners would therefore be necessary to verify whether such factor would emerge with the Italian C-BARQ. Our results also highlight how the use of a questionnaire for research—even if representing a ‘mere’ translation of a widely used tool—requires a proper validation process, including back-translation and assessment of psychometric properties in the population to which the questionnaire will eventually be applied.

## Figures and Tables

**Table 1:** Factors and item loadings resulting from the factor analysis on the Italian C-BARQ data. Items with loading values between −0.4 and 0.4 are not reported.

Factors	Variance %	Loading
*Factor 1—Stranger-directed aggression/fear*	7.663	
10. Dog acts aggressively when approached directly by an unfamiliar adult while being walked/exercised on a leash		0.799
21. Dog acts aggressively when an unfamiliar person tries to touch or pet the dog		0.780
11. Dog acts aggressively when approached directly by an unfamiliar child while being walked/exercised on a leash		0.702
28. Dog acts aggressively toward unfamiliar persons visiting your home		0.691
16. Dog acts aggressively when unfamiliar persons approach you or another member of your family away from your home		0.671
12. Dog acts aggressively toward unfamiliar persons approaching the dog while she/he is in your car (at the gas station for example)		0.613
15. Dog acts aggressively when an unfamiliar person approaches you or another member of your family at home		0.600
40. Dog acts anxious or fearful when an unfamiliar person tries to touch or pet the dog		0.509
39. Dog acts anxious or fearful when unfamiliar persons visit your home		0.465
36. Dog acts anxious or fearful when approached directly by an unfamiliar adult while away from your home		0.447
37. Dog acts anxious or fearful when approached directly by an unfamiliar child while away from your home		0.439
18. Dog acts aggressively when mailmen or other delivery workers approach your home		0.439
*Factor 2—Dog-directed fear*	5.116	
45. Dog acts anxious or fearful when approached directly by an unfamiliar dog of the same or larger size		0.824
46. Dog acts anxious or fearful when approached directly by an unfamiliar dog of a smaller size		0.779
52. Dog acts anxious or fearful when unfamiliar dogs visit your home		0.755
53. Dog acts anxious or fearful when barked, growled, or lunged at by an unfamiliar dog		0.733
*Factor 3—Owner-directed aggression*	4.138	
19. Dog acts aggressively when his/her food is taken away by a household member		0.747
31. Dog acts aggressively when you or a household member retrieves food or objects stolen by the dog		0.713
17. Dog acts aggressively when approached directly by a household member while she/he (the dog) is eating		0.675
13. Dog acts aggressively when toys, bones or other objects are taken away by a household member		0.631
9. Dog acts aggressively when verbally corrected or punished (scolded, shouted at, etc.) by you or a household member		0.421
*Factor 4—Separation-related behavior*	4.241	
56. Restlessness/agitation/pacing when left or about to be left on its own		0.657
57. Whining when left or about to be left on its own		0.653
58. Barking when left or about to be left on its own		0.578
54. Shaking, shivering or trembling when left or about to be left on its own		0.555
55. Excessive salivation when left or about to be left on its own		0.554
59. Howling when left or about to be left on its own		0.545
*Factor 5—Chasing*	3.514	
76. Dog chases or would chase squirrels, rabbits and other small animals given the opportunity		0.895
74. Dog chases or would chase cats given the opportunity		0.750
75. Dog chases or would chase birds given the opportunity		0.731
27. Dog acts aggressively toward cats, squirrels or other small animals entering your yard		0.521
*Factor 6—Dog-directed aggression*	3.024	
23. Dog acts aggressively when approached directly by an unfamiliar male dog while being walked/exercised on a leash		0.601
29. Dog acts aggressively when barked, growled, or lunged at by another (unfamiliar) dog		0.557
24. Dog acts aggressively when approached directly by an unfamiliar female dog while being walked/exercised on a leash		0.528
26. Dog acts aggressively toward unfamiliar dogs visiting your home		0.525
*Factor 7—Attachment/attention seeking*	3.975	
70. Dog tends to sit close to, or in contact with, you (or others) when you are sitting down		0.617
69. Dog tends to follow you (or other members of household) about the house, from room to room		0.608
68. Dog displays a strong attachment for one particular member of the household		0.510
72. Dog becomes agitated (whines, jumps up, tries to intervene) when you (or others) show affection for another person		0.495
71. Dog tends to nudge, nuzzle or paw you (or others) for attention when you are sitting down		0.448
73. Dog becomes agitated (whines, jumps up, tries to intervene) when you show affection for another dog or animal		0.447
*Factor 8—Trainability*	3.446	
1. Dog returns immediately when called while off leash		0.640
3. Dog obeys a “stay” command immediately		0.513
77. Dog escapes or would escape from home or yard given the chance		0.488
6. Dog is slow to learn new tricks or tasks		0.450
5. Dog is slow to respond to correction or punishment; ‘thick-skinned’		0.424
4. Dog seems to attend to or listen closely to everything the owner says or does		0.413
*Factor 9—Energy level*	3.319	
92. Dog is active, energetic, always on the go		0.534
80. Dog chews inappropriate objects		0.523
91. Dog is playful, puppyish, boisterous		0.518
*Factor 10—Non-social fear*	4.313	
38. Dog acts anxious or fearful in response to sudden or loud noises (e.g., vacuum cleaner, car backfire, road drills, objects being dropped, etc.)		0.510
48. Dog acts anxious or fearful in response to wind or wind-blown objects		0.449
41. Dog acts anxious or fearful in heavy traffic		0.420
*Factor 11—Excitability*	3.887	
65. Just before being taken for a walk		0.661
66. Just before being taken on a car trip		0.582
64. When doorbell rings		0.431
*Factor 12—Elimination problems*	3.644	
88. Dog urinates when left alone at night, or during the daytime		0.875
89. Dog defecates when left alone at night, or during the daytime		0.769
86. Dog urinates against objects/furnishings in your home		0.583
*Factor 13—Touch sensitivity*	3.179	
49. Dog acts anxious or fearful when having nails clipped by a household member		0.609
50. Dog acts anxious or fearful when groomed or bathed by a household member		0.562
51. Dog acts anxious or fearful when having his/her feet toweled by a member of the household		0.558

**Table 2:** Cronbach’s alpha values and number of items of the 13 factors on the Italian C-BARQ.

Factor	Cronbach’s α	n. of Items
1—Stranger-directed aggression/fear	0.895	12
2—Dog-directed fear	0.865	4
3—Owner-directed aggression	0.761	5
4—Separation-related behavior	0.773	6
5—Chasing	0.826	4
6—Dog-directed aggression	0.814	4
7—Attachment/attention seeking	0.739	6
8—Trainability	0.690	6
9—Energy level	0.661	3
10—Non-social fear	0.561	3
11—Excitability	0.615	3
12—Elimination problems	0.776	3
13—Touch sensitivity	0.664	3

**Table 3:** Selected demographic characteristics of the Italian sample.

Variable	Sample Size (%) or Mean ± SD
**Dog age (year)**	5.8 ± 13.8
**Frequency (>2.5%) of breeds**	
Mix breed	37.80%
Labrador retriever	10.30%
Golden retriever	4.20%
Border collie	3.70%
German shepherd	2.70%
Jack Russel terrier	2.60%
**Dog sex**	
Male	48.70%
Female	51.30%
**Dog reproductive status**	
Castrated male	29.00%
Sterilized female	68.10%
**Owner age (year)**	38.1 ± 13.4
**Owners with dog-related jobs**	
Dog trainer/educator	1.20%
Dog sitter	0.20%
Veterinarian	0.20%
Dog groomer	0.20%
**Presence of more than 1 dog in the household**	32.70%

## Data Availability

Data supporting reported results can be found at https://researchdata.cab.unipd.it/id/eprint/808 (accessed on 9 February 2023).

## Supplementary Materials

The following supporting information can be downloaded at: https://www.mdpi.com/article/10.3390/ani13071254/s1, Table S1: The administered Italian questionnaire.
