# Supplementary material for: Canine Behavioral Assessment and Research Questionnaire (C-BARQ): Validation of the Italian Translation
Source: Animals (Basel). 2023 Apr 5;13(7):1254. doi: 10.3390/ani13071254 (PMC10093636; doi:10.3390/ani13071254)
Supplement: Supplementary file 1 [file animals-13-01254-s001.zip › animals-2268997-supplementary.pdf]

Table S1: The administered Italian questionnaire.

Risposta all'addestramento
Alcuni cani sono più obbedienti e addestrabili di altri. Ripensando agli ultimi mesi, indica quanto spesso il tuo cane ha risposto come descritto in ognuna delle seguenti situazioni:
1. Quando è senza guinzaglio torna subito quando richiamato
2. Ubbidisce immediatamente al comando "seduto" *
3. Ubbidisce immediatamente al comando "resta"
4. Sembra prestare attenzione/ascoltare attentamente tutto ciò che dici e/o fai
5. Risponde lentamente a correzioni o punizioni, ha "la testa dura"
6. È lento nell'apprendere nuovi esercizi/compiti
7. È facilmente distratto da rumori, odori o dalla vista di cose interessanti *
8. Riporta o prova a riportare legnetti, palline o altri oggetti *

Aggressività
Alcuni cani di tanto in tanto esibiscono comportamenti aggressivi. Abbaiare, ringhiare e mostrare i denti sono segni tipici di moderata aggressività. Un'aggressività più grave generalmente include i seguenti comportamenti: scagliarsi contro qualcuno, mordere o tentare di mordere. Ripensando agli ultimi mesi, indica la tendenza del tuo cane a manifestare comportamenti aggressivi, in ciascuna delle situazioni descritte sotto. Scegli un valore da 0 a 4, dove 0 indica nessun segno di aggressività, e 4 la presenza di segni di aggressività elevata.
9. Quando viene corretto o punito verbalmente (rimproverato, sgridato, etc.) da te o da un altro membro del nucleo familiare
10. Quando viene avvicinato in maniera diretta da una persona adulta sconosciuta, mentre passeggia o fa attività al guinzaglio
11. Quando viene avvicinato in maniera diretta da un bambino sconosciuto, mentre è passeggia o fa attività al guinzaglio
12. Nei confronti di persone sconosciute che si avvicinano al cane quando è in macchina (per esempio, dal benzinaio
13. Quando gli vengono portati via giocattoli, ossi o altri oggetti, da un membro del nucleo familiare
14. Quando viene lavato o spazzolato da un membro del nucleo familiare *
15. Quando una persona sconosciuta si avvicina a te o ad un altro membro del nucleo familiare, in casa
16. Quando delle persone sconosciute si avvicinano a te o ad un altro membro del nucleo familiare, fuori casa
17. Quando viene avvicinato in maniera diretta da te o da un altro membro del nucleo familiare, mentre (il cane) sta mangiando
18. Quando un fattorino o il postino si avvicina a casa
19. Quando gli viene portato via il cibo da un membro del nucleo familiare
20. Quando degli estranei passano davanti a casa mentre il cane è fuori casa, in giardino/cortile *
21. Quando una persona sconosciuta cerca di toccare o accarezzare il cane
22. Quando ciclisti, gente che fa jogging, persone su skateboard o pattini passano davanti a casa mentre il cane è fuori casa, in cortile/giardino *

23. Quando viene avvicinato in maniera diretta da un cane maschio sconosciuto mentre è a passeggio al guinzaglio
24. Quando viene avvicinato in maniera diretta da un cane femmina sconosciuto mentre è a passeggio al guinzaglio
25. Quando viene guardato fisso negli occhi da un membro del nucleo familiare *
26. Nei confronti di cani sconosciuti in visita a casa vostra
27. Nei confronti di gatti, scoiattoli o altri animali che entrano nel vostro giardino
28. Nei confronti di persone sconosciute in visita a casa vostra
29. Quando un cane (sconosciuto) gli abbaia, gli ringhia o gli si scaglia contro
30. Quando un membro del nucleo famigliare familiare lo scavalca/gli passa sopra *
31. Quando tu o un altro membro del nucleo familiare vi riprendete del cibo o degli oggetti precedentemente rubati dal cane
Ci sono altre situazioni in cui il tuo cane mostra, a volte, un comportamento aggressivo? Si/no/non so
32. Nei confronti di un altro cane (conosciuto) che vive nella stessa casa (lasciare vuoto se non ci sono altri cani in casa). *
33. Quando viene avvicinato da un altro cane (conosciuto) che vive nella stessa casa mentre si trova nella sua cuccia/luogo di riposo preferita/o (lasciare vuoto se non ci sono altri cani in casa). *
34. Quando viene avvicinato da un altro cane (conosciuto) che vive nella stessa casa, mentre sta mangiando (lasciare vuoto se non ci sono altri cani in casa). *
35. Quando viene avvicinato da un altro cane (conosciuto) che vive nella stessa casa mentre mastica/gioca con un giocattolo preferito, osso, oggetto etc. (lasciare vuoto se non ci sono altri cani in casa). *

Paura e ansia
I cani a volte mostrano segni di ansia o di paura, quando sono esposti a rumori, oggetti, persone o situazioni particolari. Tipici segni di lieve o moderata paura includono i seguenti comportamenti: evitare lo sguardo, evitare l'oggetto temuto, appiattirsi a terra o rannicchiarsi con la coda bassa o tra le zampe, uggiolare o guaire, immobilizzarsi e tremare. Una paura estrema è caratterizzata da comportamenti quali: appiattirsi o rannicchiarsi in maniera esagerata, effettuare tentativi vigorosi di fuga, allontanarsi o nascondersi dall'oggetto, dalla persona o dalla situazione temuti. Ripensando agli ultimi mesi, indica la tendenza del tuo cane a mostrare paura o ansia nelle situazioni descritte in seguito. Scegli un valore da 0 a 4, dove 0 indica nessun segno di paura/ansia, e 4 indica un livello estremo di paura/ansia.
36. Quando viene avvicinato in maniera diretta da una persona adulta sconosciuta, non in casa
37. Quando viene avvicinato in maniera diretta da un bambino sconosciuto, fuori casa
38. In risposta a rumori improvvisi o forti (ad esempio: aspirapolvere, rombi di motori, martello pneumatico, oggetti che cadono accidentalmente)
39. Quando delle persone sconosciute vengono a casa vostra
40. Quando una persona sconosciuta cerca di toccarlo o accarezzarlo
41. In situazioni di traffico auto intenso
42. In risposta a oggetti strani e poco familiari, che si trovano sul marciapiedi o vicino ad esso (ad esempio sacchetti dalla spazzatura, foglie, cartacce, bandiere sventolanti, etc.) *
43. Quando viene visitato/medicato dal veterinario *
44. Durante temporali, fuochi d’artificio o eventi simili *
45. Quando viene avvicinato in maniera diretta da un cane sconosciuto della stessa taglia o più grande

46. Quando viene avvicinato in maniera diretta da un cane di taglia più piccola
47. Quando si è trovato, per la prima volta, in situazioni sconosciute (per esempio: il primo viaggio in macchina, la prima volta in ascensore, la prima visita dal veterinario) *
48. Quando c’è vento o ci sono oggetti mossi dal vento
49. Quando gli vengono tagliate le unghie da un membro del nucleo familiare
50. Quando viene spazzolato o lavato da un membro del nucleo familiare
51. Quando gli vengono asciugate le zampe da un membro del nucleo familiare
52. Quando cani sconosciuti vengono a casa vostra
53. Quando un cane sconosciuto gli abbaia, gli ringhia o gli si scaglia contro

Comportamenti relativi a momenti di separazione
Alcuni cani mostrano segni di ansia o altri comportamenti problematici, quando vengono lasciati soli, anche per periodi relativamente brevi. Ripensando agli ultimi mesi, indica quanto spesso il tuo cane ha risposto come descritto nei seguenti punti, quando è stato lasciato solo, o appena prima di essere lasciato solo.
54. Scuotersi, rabbrividire, tremare
55. Salivazione eccessiva
56. Irrequietezza/agitazione/camminare incessantemente
57. Uggiolare
58. Abbaiare
59. Ululare
60. Rosicchiare/grattare porte, finestre, pavimenti, tende, etc. *
61. Perdita di appetito *
Ci sono altre situazioni in cui il tuo cane ha paura o è ansioso? Si / No / Non so

Eccitabilità
Alcuni cani non mostrano reazioni evidenti a eventi improvvisi o potenzialmente eccitanti, né a cambiamenti nel loro ambiente; altri invece si eccitano molto alla più piccola novità. I seguenti comportamenti sono segni di una eccitabilità da lieve a moderata: allerta, dirigersi verso la situazione/oggetto nuovo, brevi episodi di abbaio. L’estrema eccitabilità è caratterizzata da una generale tendenza a reagire in maniera esagerata. Il cane eccitabile abbaia o guaisce in modo isterico al più piccolo cambiamento, corre incontro o tutto attorno a quel che lo eccita ed è difficile da calmare. Ripensando agli ultimi mesi, indica la tendenza del tuo cane a mostrare un comportamento eccitabile nelle circostanze descritte in seguito. Scegli un valore da 0 a 4, dove 0 indica un cane calmo, e 4 un cane estremamente eccitabile.
62. Quando tu o un altro membro del nucleo familiare tornate a casa dopo un breve periodo di assenza *
63. Quando gioca con te o altri membri del nucleo familiare *
64. Quando suona il citofono/campanello
65. Appena prima di essere portato fuori per una passeggiata
66. Appena prima di essere portato in giro in macchina
67. Quando arriva gente a casa *

Ci sono altre situazioni in cui il tuo cane, a volte, diventa eccessivamente agitato? Si / No / Non so

Attaccamento e ricerca di attenzione
La maggior parte dei cani è fortemente attaccata al loro proprietario, e alcuni di loro richiedono una grande quantità di attenzioni e affetto. Ripensando all'ultimo periodo, indica quanto spesso il tuo cane ha mostrato ognuno dei seguenti segni di attaccamento o richiesta di attenzione.
68. Mostra un forte attaccamento verso uno specifico membro della famiglia
69. Tende a seguire te (o altri membri del nucleo familiare) in giro per casa, di stanza in stanza
70. Tende a sedersi vicino o a contatto con te (o altri) quando siete seduti
71. Tende a dare colpetti con il naso o dare zampate per ottenere attenzione da te (o altri) quando siete seduti
72. Si agita (uggiola, salta addosso, cerca di mettersi in mezzo) se tu (o altri) mostrate affetto per un'altra persona
73. Si agita (uggiola, salta addosso, cerca di mettersi in mezzo) quando tu (o altri) mostrate affetto per un altro cane/animale

Altri comportamenti
I cani mostrano un'ampia gamma di problemi comportamentali oltre a quelli precedentemente menzionati nel questionario. Ripensando all'ultimo periodo, indica quanto spesso il tuo cane ha mostrato ognuno dei seguenti comportamenti:
74. Rincorre i gatti, o li rincorrerebbe se ne avesse la possibilità
75. Rincorre gli uccelli, o li rincorrerebbe se ne avesse la possibilità
76. Rincorre gli scoiattoli o li rincorrerebbe se ne avesse la possibilità
77. Scappa o scapperebbe da casa, o dal giardino/cortile (se ne avesse la possibilità)
78. Si rotola nelle feci di animali o in altre sostanze "puzzolenti" *
79. Mangia i propri escrementi o bisogni, o quelli di altri animali *
80. Rosicchia oggetti inappropriati
81. "Monta" oggetti, mobili o persone *
82. Elemosina cibo in continuazione quando la gente mangia *
83. Ruba cibo *
84. È ansioso o ha paura di scendere e/o salire le scale *
85. Tira eccessivamente quando è al guinzaglio *
86. Urina su mobili o altri oggetti in casa
87. Urina quando viene avvicinato, accarezzato, manipolato o preso in braccio *
88. Urina se lasciato solo di notte o di giorno
89. Defeca se lasciato solo di notte o di giorno
90. E' iperattivo e irrequieto, ha difficoltà a calmarsi *
91. Giocherellone, cucciolone, esuberante
92. Attivo, energico, deve sempre fare qualcosa

93.	Tiene lo sguardo fisso nel vuoto *
94.	Sembra voler catturare le mosche (invisibili) *
95.	Si rincorre la coda/il posteriore *
96.	Rincorre/insegue le ombre o i punti luminosi *
97.	Abbaia ostinatamente quando allarmato o eccitato *
98.	Si lecca eccessivamente *
99.	Lecca oggetti o persone eccessivamente *
100.	Mostra altri comportamenti bizzarri, strani o ripetitivi *

* Item not resulting from the factor analysis on the Italian C-BARQ data
